# Prevalence, impact and cost of multimorbidity in a cohort of people with chronic pain in Ireland: a study protocol

**DOI:** 10.1136/bmjopen-2016-012131

**Published:** 2017-01-18

**Authors:** Brian W Slattery, Laura O'Connor, Stephanie Haugh, Christopher P Dwyer, Siobhan O'Higgins, Line Caes, Jonathan Egan, Brian E McGuire

**Affiliations:** School of Psychology and Centre for Pain Research, National University Ireland Galway, University Road, Galway, Ireland

**Keywords:** Prevalence, Chronic Pain, Multimorbidity

## Abstract

**Introduction:**

Multimorbidity (MM) refers to the coexistence of two or more chronic conditions within one person, where no one condition is considered primary. As populations age and healthcare provision improves, MM is becoming increasingly common and poses a challenge to the single morbidity approach to illness management, usually adopted by healthcare systems. Indeed, recent research has shown that 66.2% of the people in primary care in Ireland are living with MM. Healthcare usage and cost is significantly associated with MM, and additional chronic conditions lead to exponential increases in service usage and financial costs, and decreases in physical and mental well-being. Certain conditions, for example, chronic pain, are highly correlated with MM. This study aims to assess the extent, profile, impact and cost of MM among Irish adults with chronic pain.

**Methods and analysis:**

Using cluster sampling, participants aged 18 years and over will be recruited from Irish pain clinics and provided an information package and questionnaire asking them to participate in our study at three time points, 1 year apart. The questionnaire will include our specially developed checklist to assess the prevalence and impact of MM, along with validated measures of quality of life, pain, depression and anxiety, and illness perception. Economic data will also be collected, including direct and indirect costs.

**Ethics and dissemination:**

Ethical approval has been granted by the Research Ethics Committee of the National University of Ireland, Galway. Dissemination of results will be via journal articles and conference presentations.

**Strengths and limitations of this study**

- The research study aims, to account for the prevalence and cost of multimorbidity in people living with chronic pain’, are novel and would provide useful information for both the applied and research communities.
- Given the cohort of participants, the sample type may arguably be considered not entirely representative of all people living with chronic pain.
- The multimorbidity checklist has been designed based on international best practice guidelines for an Irish population.
- The current study design is time consuming as ethical approval is required for each study site.

## Introduction

The guidelines for chronic disease management have traditionally taken a single disease approach,^1^ which presents a challenge for patients who have multiple, sometimes discordant chronic conditions. As such, it has been argued that a single-morbidity approach in the context of multiple health conditions typically leads to inadequate disease management.^1^ Thus, there has been a call for a more ‘holistic’ consideration of the patient and a disease management approach that focuses on multimorbidity.^1^ ^2^ Multimorbidity (MM) refers to the coexistence of two or more chronic conditions within one person, where no one condition takes precedence over another.^3^ Despite the increasing interest of healthcare practitioners in the area of MM, Marengoni *et al*^4^ note that there remains “a remarkable gap between the harmful impact of multimorbidity at the individual and societal level and the amount of scientific and clinical research devoted to this topic” (p.435).

### Prevalence of MM

There are a variety of measures deployed to assess multimorbidity (eg, Agborsangaya *et al*, 5 Britt *et al*^1^). Typically, however, prevalence research uses some form of checklist (ie, lists of chronic conditions) to assess the prevalence of MM in a given population. Most of the prevalence literature and epidemiological work in the area of MM has come from research in Canada and Australia. Prevalence rates for these countries are 19% and 37.1%, respectively.^1^ ^5^ However, epidemiological research has found prevalence estimates of MM ranging from 17% to over 90% internationally.^5^ In their Public Health Review, Boyd and Fortin^3^ concluded that approximately one out of every four adults has two or more chronic conditions, and that half of all older adults globally have three or more chronic conditions.

From an Irish perspective, relatively little research on the prevalence of MM has been conducted; however, available figures show that between 27%^6^ and 66.2%^7^ of the population have two or more chronic conditions. While there is some uncertainty regarding the exact prevalence rates of MM, it is clear that MM is becoming increasingly more common.^5^ Contributing factors to the increase in MM include ageing populations, better medical treatments, lifestyle factors and the increased prevalence of certain diseases in particular populations.^5^ ^8^

### Impact of MM: challenges for patients and health practitioners

The occurrence of MM has significant social, psychological, economic and physical implications for a person, creating a variety of management and treatment challenges.^3^ For instance, different conditions require different and sometimes incompatible treatment solutions and, as a result, multiple coexisting conditions can complicate medical treatment and affect long-term recovery. Indeed, MM can contribute to a person becoming increasingly ill compared to another person with any one of the same index diseases but without MM, and it has also been linked to higher rates of postoperative complications.^9^ Research on the impact of MM shows that it causes a decline in physical and mental functioning, is correlated with mental health issues, negatively influences quality of life, ability to work and employability and is associated with increased mortality risk, as well as longer hospital stays and higher levels of healthcare usage.^3^ ^4^ ^9^ ^10^

### MM and chronic pain

Chronic pain (CP) is defined as “an unpleasant sensory and emotional experience associated with actual or potential tissue damage, or described by the patient in terms of such damage” that persists for a period in excess of 3 months.^11^ CP is a major public health problem that can have debilitating physical, emotional, psychological and financial consequences for those individuals living with it (see Azevedo *et al*^12^; Kroenke *et al*^13^; Raftery *et al*^14^). Prevalence estimates for CP vary across studies and countries;^12–16^ one recent study found that 35.5% of the Irish population were living with CP.^14^

CP is highly correlated with MM, and is consistently identified as one of the most common conditions in those identified as having MM.^5^ For example, in one Canadian study examining the prevalence of disease combinations, 16 common disease pairs were identified, with CP appearing in six of the combinations. Further, from the five most common disease triads identified in the same study, CP was involved in three of these combinations.^5^ However, though the prevalence of CP is highly correlated with MM, little is known about the experience of chronic pain with other complex conditions.^17^ To address this issue, Butchart *et al*^17^ examined the experience and management of chronic pain for people with other chronic conditions. The researchers found that patients with CP were more likely to report decreased health than those without CP, and those with CP and comorbid heart failure or diabetes were less likely to be in employment.

Given the dearth of research examining the experience of MM where CP is a feature, as well as the high correlation and prevalence of CP and MM, it is important that research examines the prevalence and the relationship between the two more closely. While previous studies have examined the prevalence of MM in Irish samples,^7^ no previous Irish study has examined the prevalence of MM in a population of people with CP.

### Aims of the current research

1. To determine the prevalence, impact and cost of MM in a cohort of people in Ireland who live with chronic pain.
2. To identify the nature and profile of MM in which chronic pain is a central feature.
3. To develop a predictive model of multimorbid disability in a population of people with chronic pain.
4. To chart the developmental trajectory of MM in a sample of people with chronic pain.

## Method

### Design

A prospective cohort study with three time points (1 year apart) assessing the prevalence of MM in a cohort of people with chronic pain will be employed.

### Data collection and sample size

#### Inclusion criteria

All participants of this study must be over 18 years of age and experiencing chronic non-cancer pain (according to the International Association for the Study of Pain definition). Individuals with terminal illness, severe mental illness or cognitive impairment that would prevent adequate understanding and participation in the study will be excluded.

#### Recruitment

Recruitment will be carried out through Irish pain clinics. Staff in the pain clinics will inform patients of the study. Participants will be identified via the patient records from each of the 16 pain clinics in the Republic of Ireland; a list of patients who have visited each clinic over the previous 18 months will be requested. Each patient will be given an identifier by one of the members of the research team (L'OC), and another member of the research team (SH) will employ Stata V.13.1 (Stata. College Station, Texas: StataCorp LP 2013) to randomly select 150 participants from each clinic. These participants will be given the survey packs containing the study information sheets, consent forms, multimorbidity checklist and questionnaires by the research team. The participants will be given the option to post their completed packs to the NUI Galway Centre for Pain Research using a stamped addressed envelope provided. In addition, the pack will contain a link to our website where the participants will be able to complete the survey online should they prefer this method to the postal system.

#### Sample size

Sample size was calculated using the equation proposed for prevalence research by Naing *et al*.^18^ To calculate the sample size for a prevalence study, the expected prevalence is required. Based on previous research,^6^ ^7^ ^14^ ^19^ the expected prevalence of MM in a population of people with CP is approximately between 22% and 54%. Nicholl^20^ notes that if there is a doubt about the prevalence total in a given population, researchers should err towards assuming a 50% prevalence rate as it will yield a larger sample size. Therefore, using Naing *et al*'s^18^ equation and predicting that 50% of our sample may exhibit MM, CIs set at 0.95 and the degree of precision (d)=0.05 produced a sample size of 384. Using a predicted response rate of 40%, based on previous Irish prevalence research in the area of chronic pain, the sample size was calculated as 960. Since we are sampling from Irish pain clinics, the design effect of using a cluster randomised trial must be taken into account; however, we were unable to identify a suitable intracluster correlation coefficient. Therefore, as demonstrated in a similar study on prevalence,^14^ a median value of 0.01 was used. Adjusting for this, with an average of 150 patients per cluster, gives a design effect of 2.49 and a sample of 2391. A target sample of 2400 will be recruited, 150 patients from each of the 16 Irish pain clinics.

### Measures

#### Sociodemographic and health information

Participants will be asked to supply details regarding age, gender, relationship status, highest educational attainment, occupational status (working full-time, working part-time, retired, unemployed, occupied with home duties or other) and their occupation, to determine socioeconomic status, as well as duration of chronic conditions, site(s) of chronic pain and cause of chronic pain. Some details about previous and current medical and alternative treatments will also be collected.

#### Primary measure

The main focus of this study is to assess the point prevalence of MM in a cohort of people with chronic pain in Ireland. To that end, a specific disease count measure was developed.

##### Background to current MM checklist

As Diederichs, Berger and Bartels^10^ highlight, there is no ‘gold standard’ measure of MM. Several measures of MM exist and have been developed for a variety of reasons, including different definitions of MM, different purposes for measuring MM, different required or available resources for data collection and the type of data available.^7^ ^21^ Moreover, there are no definitive criteria for the selection of chronic conditions that qualify for MM and, therefore, no standardised list of the number and type of diseases to be included in a MM measure.^10^ In a review of the literature, de Groot, Beckerman, Lankhorst and Bauter^22^ found that there were 13 common measures of MM: 12 of these were disease indexes and one was a disease count.

Researchers who are interested in tallying the number of conditions that occur in patients as an outcome, or those examining the prevalence of MM, primarily use disease counts (eg, Bayliss *et al*^21^). To develop a disease count measure, researchers typically select and include the most common conditions found in the targeted population. For example, Bayliss *et al*^21^ reviewed the literature and selected the 25 most common chronic conditions for a US sample to include in their measure. They developed a subjective scale or disease count, where participants marked which diseases they had and then rated them in terms of severity (ie, how much each one affected their daily functioning).

Although different viewpoints exist regarding what conditions to include and how they should be selected for an MM disease count measure, guidelines^21^ ^23^ have been proposed to address these issues, which are based on work yielded from systematic reviews in the area.

##### Guidelines for developing an MM checklist

Fortin *et al*^23^ proposed an operational definition of MM, whereby two or more diseases should be present in an individual and meet the diagnostic criteria for two separate areas of the Cumulative Illness Rating Scale (CIRS).^24^ The CIRS is a measure that weights the severity of MM. It is divided into sections based on 14 different organ systems. These organ systems are as follows: (1) Cardiac, (2) Vascular, (3) Haematopoietic, (4) Respiratory, (5) Eyes, Ears, Nose, Throat and Larynx, (6) Upper gastrointestinal (GI), (7) Lower GI, (8) Hepatic, (9) Renal, (10) Genitourinary, (11) Musculoskeletal, (12) Neurological, (13) Endocrine/Metabolic and Breast, and (14) Psychiatric. For a person to be considered to have a diagnosis of MM, chronic disease must be present in at least two different sections or organ systems. However, it is not necessary for a disease count to list either all conditions or all CIRS body systems. Fortin *et al*^25^ recommend a minimum of seven conditions and argued that any list of conditions included in a MM prevalence study should reflect the most common conditions in the population to be studied.

##### Process for developing the current MM checklist

In line with Fortin *et al*'s^26^ recommendations, the conditions included in the current disease count questionnaire were obtained from two large-scale national reports on the Irish population more widely^25^ ^26^ and four Irish research studies that had investigated the prevalence of MM and had compiled lists of conditions to examine.^6^ ^19^ ^26^ ^27^ To ensure that the list of conditions met international best practice recommendations,^21^ ^23^ in terms of which conditions to include in an MM disease count list, we then combined our study with one international study that examined MM in people living with CP.^5^ We also examined two of the most recent systematic reviews, which outline the most common conditions included in MM studies and contain recommendations for the type of conditions to be included in MM checklists.^10^ ^28^ Following this, two healthcare practitioners were consulted and provided feedback on which conditions to include, and one clinician, who is an expert in chronic pain, reviewed the entire MM checklist. From this review process, the three additional categories of conditions included were as follows: renal disorders, hepatic disorders and headache disorders. Subsequently, we collapsed the conditions from this developmental process with respect to the CIRS organ system domains and removed any duplicate conditions, leaving us a total of 34 conditions across 10 organ domains (see table 1). We also added category options (eg, ‘Other cardiac conditions’) to ensure that useful data could be collected on conditions that did not appear on the list, as well as a final ‘Any other condition not listed’ category.

**Table 1:** Source and summary of conditions included in the Multimorbidity Checklist

CIRS domain	Study and included conditions									Included MM Checklist conditions
	Teljeur *et al*^27^	Naughton *et al*^6^	Household Quarterly Report 2010	CARDI 2011/Savva *et al* 2011 unpublished manuscript	TILDA 2011 Report: Fifty plus in Ireland	Diederichs *et al*^10^ (systematic review)	Sinnige *et al*^28^(systematic review)	Agborsangaya *et al*^5^	Sinnott *et al*^19^	
Cardiac	Heart disease	Hypertension/heart failure, arrhythmias	Heart Attack, heart failure	Angina, heart attack	Cardiovascular disease (angina, heart attack, heart failure)	Chronic ischaemic heart disease, arrhythmia, insufficiency, infarction	Heart disease, heart failure, attack, angina (coronary artery disease)		Prior heart attack, angina, heart failure, aortic aneurysm, other cardiac disease, peripheral vascular disease,	Angina, arrhythmia, heart failure, heart attack, other
Vascular	Hypertension	Hypertension	Hypertension		Hypertension	Hypertension	Hypertension	Hypertension	Hypertension	Hypertension
Haematopoietic	High cholesterol, hyperlipidaemia,	Hypercholesterolaemia	High cholesterol				Lipid metabolism disorders	High cholesterol		High cholesterol
Respiratory	Chest/lung disease, asthma	Respiratory conditions		Asthma, COPD	Respiratory disease (eg, bronchitis or emphysema)	COPD	COPD, asthma		Asthma, bronchitis	Asthma, bronchitis, emphysema, COPD, other
Eyes, ears, nose, throat and larynx		Glaucoma			Eye disease (eg, glaucoma, age-related macular degeneration, cataracts)					Glaucoma, other
UPPER GI (oesophagus, stomach, duodenum)							Gastrointestinal disease			Gastrointestinal disease, Other GI conditions
LOWER GI										
*Hepatic										Liver disease (eg, hepatitis)
*Renal										Kidney disease (eg, chronic kidney disease)
Genitourinary									Urinary incontinence	Urinary incontinence, other
Musculoskeletal	Arthritis	Musculoskeletal conditions, Pagets/osteoporosis, Rheumatological conditions	Osteoporosis		Pain, arthritis, osteoporosis		Arthritis, osteoarthritis, osteoporosis, chronic back or neck disorder	Arthritis	Chronic back pain, osteoarthritis, osteoporosis, rheumatoid arthritis	Back pain or problem, neck pain or problem, osteoporosis, osteoarthritis, rheumatoid arthritis, other
*Headache disorders										Headache disorder (eg, migraine, cluster headaches, tension headaches)
Neurological		Parkinson's disease, dementia	Stroke	Stroke	Stroke/TIA	Stroke	Dementia, cerebrovascular disease/stroke		Stroke	Stroke, TIA, Dementia, Other CNS conditions
Endocrine/metabolic and breast	Diabetes, hypothyroidism, obesity	Diabetes, thyroid disorders		Diabetes	Diabetes	Diabetes	Diabetes, thyroid disease, obesity	Diabetes, obesity	Thyroid disease, diabetes	Diabetes (type 1), Diabetes (type 2), hypothyroidism, obesity Other
Psychiatric	Depression	Psychiatric disorders, anxiety			Depression, anxiety	Depression	Depression	Depression/anxiety	Anxiety, depression	Depression, anxiety, other
Other conditions:		Cancer		Cancer		Cancer	Cancer	Cancer	Cancer	Any cancer in the past 5 years
		Epilepsy, gout, sleep disturbance						Sleep apnoea		Epilepsy, gout, chronic sleep disturbance

This table summarises the process of integrating previous multimorbidity studies and their listed conditions with the body system (CIRS domain) approach to develop the final list of conditions that appear in our multimorbidity tool (see the rightmost column). *Conditions included, as a result of advice from a healthcare practitioner.

CIRS, Cumulative Illness Rating Scale; MM, Multimorbidity.

COPD, chronic obstructive pulmonary disease; CNS, central nervous system; GI, gatroinstestinal; TIA, transient ischaemic attack.

##### Structure of MM checklist and operational definition of MM

Based on Fortin *et al*'s^8^ suggestion, a condition will be deemed suitable for inclusion as a MM when it meets one or both of the following criteria; a formal diagnosis has been provided by a doctor, and/or a person is receiving prescribed medication for the particular condition. To ensure that participants understand what each condition is, a lay definition derived from a medical definition for each condition (see US National Library of Medicine MeSH database^29^) will be provided (see online supplementary appendix A). Furthermore, similar to Bayliss *et al*,^21^ who created a subjective survey disease count measure of MM, the current measure will include a rating scale (from 1 to 5; 1 being least impactful and 5 being most impactful) measuring the impact that each condition has on their daily functioning. The inclusion of this rating scale will enable the research to identify which chronic conditions have more of an impact on daily functioning and, indeed, which disease combinations have more of a cumulative impact on daily functioning.

10.1136/bmjopen-2016-012131.supp1 supplementary data

#### Secondary measures

A number of secondary measures will be included to provide an accurate representation of the impact of MM and CP on participants. The measures outlined below were chosen to quantify the prevalence and impact of MM for people living with CP and were based on inclusion in previous chronic pain and MM prevalence research.^14^

##### Health-related quality of life

The Medical Outcomes Short Form-12 (SF-12)^30^ will be used to assess health-related quality of life. The SF-12 is a general measure of health-related quality of life that has been used and validated with European populations.^31^ It gathers information across 8 health domains: general health, physical functioning, emotional role limitation, physical role limitation, mental health, bodily pain, vitality and social functioning. According to the norm-based method recommended by the test author, these items are scored to produce a mental component summary and physical component summary of health-related quality of life.^30^ Lower scores on either of these scales are indicative of a lower quality of life. Irish population norms are available^32^ and will be used for comparison with the present sample. The SF-12 has been used as a measure of health-related quality of life previously within CP research and MM research.^20^ ^33^ ^34^

##### Depression and anxiety

Depression will be measured using the Patient Health Questionnaire-9 item (PHQ-9). The PHQ-9 is a widely used and well validated measure of depression^13^ and it has been used with people living with chronic conditions.^35^ It contains 9 items that relate to the Diagnostic and Statistical Manual Fourth Edition (DSM IV) criteria for depression. The items are scored on a four-point Likert scale ranging from 0 ‘not at all’ to 3 ‘nearly every day’. The higher the score on the PHQ-9, the more symptom criteria a person meets. A cut-off score of above 10 indicates moderate depression and a score of above 15 indicates a clinical ‘case’ of moderately severe depression.

Anxiety will be measured using the generalised anxiety disorder 7-item (GAD-7). The GAD-7 is a validated and standardised measure of anxiety^36^ and has been recommended for use in CP studies.^37^ It is a 7-item questionnaire that presents items relating to how often over the past couple of weeks a person has felt bothered by each of the DSM IV symptom criteria for GAD. Items are scored on a four-point Likert scale ranging from 0 ‘not at all’ to 3 ‘nearly every day’. A higher overall score represents greater symptom severity.

##### Pain severity and disability

Pain-related severity and disability will be measured by the Chronic Pain Grade Questionnaire,^38^ commonly used in pain research. The Chronic Pain Grade Questionnaire categorises pain severity into one of four grades based on two dimensions: intensity and disability, depending on pain experiences in the previous 3–6 months. It contains seven items which can be completed by self-report, and includes questions both about the pain itself and its impact on daily functioning.

##### Pain intensity and interference

Intensity of pain and the degree of interference in the participant's life will be measured by the Brief Pain Inventory, specifically the short form of the tool.^39^ This includes nine items, to be completed by self-report, and asks about pain both now and over time. Two scores are given: pain severity (out of 40) and pain interference (out of 70). Higher scores indicate greater pain severity and interference.

##### Multimorbidity Illness Perceptions Scale

The Multimorbidity Illness Perceptions Scale (MULTIPleS)^40^ was developed to measure patient illness perceptions in the presence of MM. The MULTIPleS is a 22-item questionnaire. Each item has a Likert scale that runs from 0 to 3, where ‘0’ indicates that a person ‘strongly disagrees’ with an item and ‘3’ indicates that a person ‘strongly agrees’ with an item. Overall, the 22 items comprise five subscales: emotional representation, treatment burden, prioritising conditions, causal links and activity limitations. The MULTIPleS is a relatively new scale, so it has not yet been used as a measure in clinical research. However, Gibbons *et al*^40^ found that the scale provided a good fit to the Rasch model and demonstrated evidence of reliability and validity for each of the subscales.

### Economic evaluation

The economic evaluation will be based on a number of questions relating to usage of healthcare services and financial costs to the participant (similar to Raftery, Ryan & Normand^41^). More specifically, we will examine costs that fall on the health and social care services by recording hospitalisations (frequency and duration), outpatient appointments, accident and emergency appointments, types and amounts of benefits received per month, community services used (eg, general practitioner and home help), and medication type, dosage and frequency. These services/products will be translated into unit cost data for Ireland and provide an estimate of the cost of MM where CP is a feature for the health service. Furthermore, we will calculate indirect costs incurred personally by each individual with MM and their family. These will include expenditure for treatments and medications not paid for by the state, and the travel and wait time costs associated with availing of health services. Opportunity costs will also be calculated by quantifying work absenteeism or reduced employment due to MM. To generate these data, information on wages will be collected; however, should collection of this information not be possible, we will extrapolate income from age, education and work type.

### Risk of bias

To reduce the risk of participant selection bias, one researcher (LO'C) will give each potential participant a unique identifier and another member of the research team (SH) will use STATA V.13.1 to randomly select participants. Furthermore, responders will be compared to non-responders to assess and ensure that there is no response bias between those who actively participate and those who do not,

### Statistical analyses

Graphical (eg, box plots, labelled scatter plots and case profile plots) and numerical summaries (means, medians, SDs and IQRs) will be provided for all variables. A χ^2^ test will be used to evaluate the relationship between gender and number of conditions. ORs will be calculated for risk factors of MM. Factors associated with MM will be analysed using univariate multiple regression and hierarchical regression will be employed to examine the relationships among the number and type of conditions and the outcome variables (ie, depression, anxiety, quality of life, illness perceptions and severity of pain, for example). All analyses will be conducted using SPSS V.22.

### Data monitoring and management

This study will collect non-identifying, minimally invasive information and as such does not require a formal data monitoring committee. All information collected will be stored securely at the research site. Paper documents will be kept in locked cabinets, and electronic data will be stored on password-protected databases that can only be accessed by the research team.

### Dissemination

Findings of the study will be disseminated in peer-reviewed publications following the data analysis. Researchers will also present the results at conferences. The research programme website will be regularly updated with news about the study to facilitate dissemination to the general public.
