# Supplementary material for: Prevalence, impact and cost of multimorbidity in a cohort of people with chronic pain in Ireland: a study protocol
Source: BMJ Open. 2017 Jan 18;7(1):e012131. doi: 10.1136/bmjopen-2016-012131 (PMC5253562; doi:10.1136/bmjopen-2016-012131)
Supplement: supplementary data [file bmjopen-2016-012131supp_appendix.pdf]

Appendix A: Included conditions

The table below describes the conditions included in our study in more detail, using the MeSH descriptions for each condition for clarity. Each condition also lists a lay description, which will be used for greater clarity in the final questionnaire.

Condition	MeSH Description	Lay Description
**Angina**	The symptom of paroxysmal pain consequent to MYOCARDIAL ISCHEMIA usually of distinctive character, location and radiation. It is thought to be provoked by a transient stressful situation during which the oxygen requirements of the MYOCARDIUM exceed that supplied by the CORONARY CIRCULATION.	Angina is chest pain or discomfort where your heart muscle does not get enough blood, which may feel like pressure or a squeezing pain in your chest, indigestion, or pain in your shoulders, arms, neck, jaw or back. There are three types of angina: stable, unstable and variant.
**Arrhythmia**	Any disturbances of the normal rhythmic beating of the heart or MYOCARDIAL CONTRACTION. Cardiac arrhythmias can be classified by the abnormalities in HEART RATE, disorders of electrical impulse generation, or impulse conduction.	Cardiac arrhythmia, also known as cardiac dysrhythmia or irregular heartbeat, is a group of conditions where the heartbeat is irregular, too fast, or too slow.
**Heart Failure**	A heterogeneous condition in which the heart is unable to pump out sufficient blood to meet the metabolic need of the body.	A condition which occurs when the heart is unable to pump sufficiently to meet the body's needs.
**Heart Attack**	NECROSIS of the MYOCARDIUM caused by an obstruction of the blood supply to the heart (CORONARY CIRCULATION).	A heart attack occurs when blood stops flowing to part of the heart causing damage to the heart muscle. Also known as myocardial infarction (MI) or acute myocardial infarction (AMI),
**Other Cardio**	Aneurysm, atherosclerosis, peripheral artery disease, pericardial disease, cardiomyopathy or other	
**Hypertension**	Persistently high systemic arterial BLOOD PRESSURE. Based on multiple readings (BLOOD PRESSURE DETERMINATION), hypertension is currently defined as when SYSTOLIC PRESSURE is consistently greater than 140 mm Hg or when DIASTOLIC PRESSURE is consistently 90 mm Hg or more.	Blood pressure is the force of blood pushing against the walls of the arteries as the heart pumps blood. Hypertension or high blood pressure refers to this pressure rising and remaining high.
**High Cholesterol**	A condition with abnormally high levels of CHOLESTEROL in the blood. It is defined as a cholesterol value exceeding the 95th percentile for the population.	The presence of high levels of cholesterol in the blood (also called hypercholesterolemia or dyslipidaemia).
**Hepatic conditions**	Liver disease, cirrhosis, hepatitis, other liver conditions or other	
**Asthma**	A form of bronchial disorder with three distinct components: airway hyper-responsiveness (RESPIRATORY HYPERSENSITIVITY), airway INFLAMMATION, and intermittent AIRWAY OBSTRUCTION. It is characterized by spasmodic contraction of airway smooth muscle, WHEEZING, and dyspnea (DYSPNEA, PAROXYSMAL).	A common chronic disease of the airways which obstructs normal breathing with wheezing and coughing.
**Bronchitis**	A subcategory of CHRONIC OBSTRUCTIVE PULMONARY DISEASE. The disease is characterized by hypersecretion of mucus accompanied by a	Chronic bronchitis is defined as a cough that occurs every day with sputum production and that lasts for at least 3 months.

	chronic (more than 3 months in 2 consecutive years) productive cough. Infectious agents are a major cause of chronic bronchitis.	
**Emphysema**	Enlargement of air spaces distal to the TERMINAL BRONCHIOLES where gas-exchange normally takes place. Pulmonary emphysema can be classified by the location and distribution of the lesions.	An inflammatory response in the lungs resulting in narrowing of the small airways and breakdown of lung tissue
**Chronic Obstructive Pulmonary Disease (COPD)**	A disease of chronic diffuse irreversible airflow obstruction. Subcategories of COPD include CHRONIC BRONCHITIS and PULMONARY EMPHYSEMA. Enlargement of air spaces distal to the TERMINAL BRONCHIOLES where gas-exchange normally takes place. This is usually due to destruction of the alveolar wall. Pulmonary emphysema can be classified by the location and distribution of the lesions.	A type of obstructive lung disease characterized by chronically poor airflow
**Other Respiratory**	Pulmonary edema, embolism, obstructive sleep apnea, tuberculosis or other	
**Glaucoma**	An ocular disease, occurring in many forms, having as its primary characteristics an unstable or a sustained increase in the intraocular pressure which the eye cannot withstand without damage to its structure or impairment of its function.	A group of ocular (eye) disorders that result in optic nerve damage, often associated with increased fluid pressure in the eye
**Other Eyes, Ears, Nose, and Throat**	Hearing loss, tinnitus, meniere's disease, sinus conditions, obstructive sleep apnea, vocal cord disorders or other	
**Gastrointestinal disease**	Disease in any part of the GASTROINTESTINAL TRACT or the accessory organs (LIVER; BILIARY TRACT; PANCREAS).	Disease involving the gastrointestinal tract, namely the oesophagus, stomach, small intestine, large intestine and rectum, and the accessory organs of digestions, the liver (e.g., hepatitis), gallbladder, and pancreas (e.g., diabetes).
**Other GI conditions**	IBS, colitis, GERD or other	
**Renal conditions**	Kidney disease, renovascular disease, amyloidosis, lupus nephritis, or other	
**Back Pain or Problem**	Chronic pain located in the posterior regions of the THORAX; LUMBOSACRAL REGION; or the adjacent regions.	Pain felt in the back that usually originates from the muscles, nerves, bones, joints or other structures in the spine.
**Neck pain or problem**	Discomfort or more intense forms of pain that are localized to the cervical region. This term generally refers to pain in the posterior or lateral regions of the neck.	Pain felt in the neck
**Other Pain Condition**	Pain in any other location or a more general pain condition	
**Osteoporosis**	Reduction of bone mass without alteration in the composition of bone, leading to fractures. Primary osteoporosis can be of two major types: postmenopausal osteoporosis (OSTEOPOROSIS, POSTMENOPAUSAL) and age-related or senile osteoporosis.	A disease where decreased bone strength increases the risk of a broken bone
**Osteoarthritis**	A progressive, degenerative joint disease, the most common form of arthritis, especially in older persons.	Osteoarthritis (also known as degenerative arthritis, degenerative joint disease, or osteoarthrosis) is a type

		of joint disease that results from breakdown of joint cartilage and underlying bone
**Rheumatoid arthritis**	A chronic systemic disease, primarily of the joints, marked by inflammatory changes in the synovial membranes and articular structures, widespread fibrinoid degeneration of the collagen fibers in mesenchymal tissues, and by atrophy and rarefaction of bony structures.	A chronic, systemic inflammatory disorder that primarily affects joints
**Other muskuloskeletal**	Carpal tunnel syndrome, tendonitis, joint disorders, other bone/muscle/joint pain, repetitive strain injury or other	
**Stroke**	A group of pathological conditions characterized by sudden, non-convulsive loss of neurological function due to BRAIN ISCHEMIA or INTRACRANIAL HEMORRHAGES. Stroke is classified by the type of tissue NECROSIS, such as the anatomic location, vasculature involved, etiology, age of the affected individual, and hemorrhagic vs. non-hemorrhagic nature.	Stroke (also known as cerebrovascular accident (CVA), cerebrovascular insult (CVI), or brain attack) is when poor blood flow to the brain results in cell death. There are two main types of stroke: ischemic due to lack of blood flow and hemorrhagic due to bleeding.
**TIA (Transient Ischaemic Attack)**	Brief reversible episodes of focal, non-convulsive ischemic dysfunction of the brain having a duration of less than 24 hours, and usually less than one hour, caused by transient thrombotic or embolic blood vessel occlusion or stenosis	Often referred to as mini-strokes - A short, temporary episode of neurologic dysfunction caused by loss of blood flow.
**Dementia**	An acquired organic mental disorder with loss of intellectual abilities of sufficient severity to interfere with social or occupational functioning. The dysfunction is multifaceted and involves memory, behavior, personality, judgment, attention, spatial relations, language, abstract thought, and other executive functions. The intellectual decline is usually progressive, and initially spares the level of consciousness.	A broad category of brain diseases that cause a long term and often gradual decrease in the ability to think and remember such that a person's daily functioning is affected
**Headache disorders**	E.g. migraine, cluster headache, tension headaches	
**Other CNS/Neuro conditions**	Bell's palsy, dyskinesia, multiple sclerosis, motor speech disorders, Parkinson's, motor neurone disease or other	
**Diabetes (Type 1),**	A subtype of DIABETES MELLITUS that is characterized by INSULIN deficiency. It is manifested by the sudden onset of severe HYPERGLYCEMIA, rapid progression to DIABETIC KETOACIDOSIS, and DEATH unless treated with insulin. The disease may occur at any age, but is most common in childhood or adolescence.	Lack of insulin resulting from the autoimmune destruction of the insulin-producing cells in the pancreas which leads to increased blood and urine glucose, where administration of insulin is essential for survival
**Diabetes (Type 2),**	A subclass of DIABETES MELLITUS that is not INSULIN-responsive or dependent (NIDDM). It is characterized initially by INSULIN RESISTANCE and HYPERINSULINEMIA; and eventually by GLUCOSE INTOLERANCE; HYPERGLYCEMIA; and overt diabetes. Type II diabetes mellitus is no longer considered a disease exclusively found in adults. Patients seldom develop KETOSIS	A metabolic disorder that is characterized by high blood sugar in the context of insulin resistance and relative lack of insulin, initially managed by increasing exercise and dietary changes

	but often exhibit OBESITY.	
**Hypothyroidism**	A syndrome that results from abnormally low secretion of THYROID HORMONES from the THYROID GLAND, leading to a decrease in BASAL METABOLIC RATE.	A common endocrine disorder in which the thyroid gland does not produce enough thyroid hormone. It can cause a number of symptoms, such as tiredness, poor ability to tolerate cold, and weight gain
**Other Endocrine/Metabolic**	Cushing's syndrome, cystic fibrosis, hyperthyroidism, PCOS, or other	
**Depression**	An affective disorder manifested by either a dysphoric mood or loss of interest or pleasure in usual activities. The mood disturbance is prominent and relatively persistent.	Clinical depression (also known as major depressive disorder (MDD), major depression, unipolar depression, or unipolar disorder; or as recurrent depression in the case of repeated episodes) is a mental disorder characterized by a pervasive and persistent low mood that is accompanied by low self-esteem and by a loss of interest or pleasure in normally enjoyable activities
**Anxiety**	Persistent and disabling ANXIETY. - Feeling or emotion of dread, apprehension, and impending disaster	Anxiety disorders are a group of mental disorders characterized by feelings of anxiety and fear
**Other psychiatric**	Eating disorders, obsessive compulsive disorders, schizophrenia, bipolar disorder, pervasive developmental disorder, substance abuse or other	
**Any cancer in the past 5 years**	New abnormal growth of tissue. Malignant neoplasms show a greater degree of anaplasia and have the properties of invasion and metastasis, compared to benign neoplasms.	Cancer, also known as a malignant tumour or malignant neoplasm, is a group of diseases involving abnormal cell growth with the potential to invade or spread to other parts of the body
**Epilepsy**	A disorder characterized by recurrent episodes of paroxysmal brain dysfunction due to a sudden, disorderly, and excessive neuronal discharge. Epilepsy classification systems are generally based upon: (1) clinical features of the seizure episodes (e.g., motor seizure), (2) etiology (e.g., post-traumatic), (3) anatomic site of seizure origin (e.g., frontal lobe seizure), (4) tendency to spread to other structures in the brain, and (5) temporal patterns (e.g., nocturnal epilepsy).	A group of neurological disorders characterized by epileptic seizures. Epileptic seizures are episodes that can vary from brief and nearly undetectable to long periods of vigorous shaking
**Gout**	Hereditary metabolic disorder characterized by recurrent acute arthritis, hyperuricemia and deposition of sodium urate in and around the joints, sometimes with formation of uric acid calculi.	Usually characterized by recurrent attacks of acute inflammatory arthritis—a red, tender, hot, swollen joint.
**Genitourinary conditions**	Urinary incontinence, urinary frequency, chronic UTI, or other	
**Sleep Disorder**	Conditions characterized by disturbances of usual sleep patterns or behaviors. Sleep disorders may be divided into three major categories: DYSSOMNIAS (i.e. disorders characterized by insomnia or hypersomnia), PARASOMNIAS (abnormal sleep behaviors), and sleep disorders secondary to medical or psychiatric disorders	Sleep disorders are broadly classified into dyssomnias, parasomnias, circadian rhythm sleep disorders involving the timing of sleep, and other disorders including ones caused by medical or psychological conditions and sleeping sickness.
**Obesity**	To be determined by calculating BMI	

**Any other conditions**	Please list any other conditions that affect you but do not appear on this list:
